# Muscle Fat Content Is Strongly Associated With Hyperuricemia: A Cross-Sectional Study in Chinese Adults

**DOI:** 10.3389/fendo.2022.935445

**Published:** 2022-06-28

**Authors:** Ningxin Chen, Tingting Han, Hongxia Liu, Jie Cao, Wenwen Liu, Didi Zuo, Ting Zhang, Xiucai Lan, Xian Jin, Yurong Weng, Yaomin Hu

**Affiliations:** Department of Geriatrics, Renji Hospital, School of Medicine, Shanghai Jiaotong University, Shanghai, China

**Keywords:** skeletal muscle fat index, myosteatosis, uric acid, hyperuricemia, Chinese adults

## Abstract

Studies have indicated that the skeletal muscle mass and strength was related to serum uric acid (UA), but there is a lack of research on the association of skeletal muscle fat content with UA. The purpose of this cross-sectional study is to investigate the correlation of skeletal muscle fat index (SMFI) and hyperuricemia (HUA) in Chinese adults. 500 subjects (306 men and 194 women) were included in the study. The participants were divided into four groups according to SMFI quartiles. Pearson’s correlations between SMFI and metabolic variables were calculated. Logistic regression analysis was used to estimate the association between the quartiles of SMFI and risk of hyperuricemia. UA showed a positive association with SMFI after adjusted for BMI, age and gender. A significant association between the SMFI and risk of HUA was found, the OR for HUA was 2.79 (95% CI 1.18-6.59, p<0.05) in Q2, 2.41(95% CI 1.00-5.81, p<0.05) in Q3, and 2.63 (95% CI 1.03-6.72, p<0.05) in Q4, after adjusted for BMI. In conclusion, the SMFI was significantly associated with the level of serum UA, and the higher SMFI may indicate a higher risk of HUA, independent of BMI.

## Introduction

Uric acid (UA) is a metabolic end-product of purine compounds, normal serum UA level is controlled by the balance between synthesis and excretion of urate. Clinical and epidemiological studies have suggested associations between increased UA level with obesity, hypertension, insulin resistance, dyslipidemia, liver dysfunction, renal dysfunction and gouty arthritis (1–8), it was indicated that UA is a mediator of metabolic dysfunction (9, 10), it also reflects prognosis of some diseases. In recent decades, Hyperuricemia (HUA) has become a common metabolic disorder worldwide. A systematic review and meta-analysis have shown that the pooled prevalence of HUA was 13.3% in Mainland China from 2000 to 2014 (11). A recent study among 374,506 Chinese subjects, whose data were collected from 2012 to 2018, showed the overall prevalence of HUA is 14.8%, it was suggested the disease burden of HUA is increasing (12).

In recent years, the concept of “muscle health” has drawn increasing attention, and there are many studies suggested that disrupted “muscle health” was associated with metabolic disease, cardiovascular disease and prognosis of tumor. Several studies have reported that serum UA levels might be associated with skeletal muscle mass and/or strength, but provided conflicting results. Beavers’s study showed that increased serum UA was significantly related to reduced skeletal muscle mass (13). Tanaka et al. found that higher UA serum levels were correlated with reduced muscle mass in men with type 2 diabetes mellitus (T2DM) (14). A cross-sectional study among Chinese adults over 45 years suggested that a specific range of serum UA levels was positively associated with hand grip strength (15). Another study showed that in the oldest higher uric acid serum levels are associated with better muscle function (16).

A high muscle fat content (often known as myosteatosis) may play a key role in the risk of metabolic abnormalities. Studies have indicated that myosteatosis is associated with insulin resistance, type 2 diabetes mellitus (T2DM), dyslipidemia and cardiometabolic diseases (17, 18), and there was also a link between skeletal muscle density and biomarkers of inflammation, such as CRP, IL-6, TNF-α, adiponectin and leptin (19). Myosteatosis has also been identified as a risk factor for decreased muscle strength, osteoporotic fractures, and reduced physical function (20, 21).

However, there is a lack of study focusing on the correlation between HUA and skeletal muscle fat content. The aim of the present study was to investigate the relationship between skeletal muscle fat index (SMFI), which was evaluated by CT, with serum UA level.

## Material and Methods

### Design and Participants

The observational and correlational study was carried out in the Department of Geriatrics of Renji Hospital between December 2020 and November 2021. Participants included in the study met the following criteria: underwent a health checkup, underwent a chest CT scan and over 18 years of age. The exclusion criteria were as follows: serious systemic diseases such as heavy infection, cancer, or severe liver and renal insufficiency, neuromuscular disease, subjects with incomplete data. The study included 500 patients (306 men and 194 women) finally. The study was approved by the Ethical Committee of Renji Hospital (approval no. KY2021-071-B). The study was performed in accordance with the Declaration of Helsinki.

### Anthropometric and Biochemical Measurements

Body height, weight was measured by trained survey personnel. Total cholesterol (TC), triglyceride (TG), high-density lipoprotein cholesterol (HDL-C), low-density lipoprotein cholesterol (LDL-C), alanine aminotransferase (ALT), aspartate aminotransferase (AST), creatinine (Cr) and uric acid (UA) were measured after an 8-hour overnight fast, serum analyses were performed using Hitachi 7600-110 and Hitachi 7020 automatic analyzer (Hitachi, Tokyo, Japan). The body mass index (BMI) was calculated as weight (kg) divided by height squared (m^2^).

### Definition of HUA

According to Chinese practice guideline for patients with hyperuricemia/gout, subjects with serum uric acid ≥420 μmol/l were defined as HUA in men and ≥360 μmol/l were defined as HUA in women (22).

### Muscle Analysis

Skeletal muscle and density were calculated from the chest CT scan at thoracic vertebra 12 (T12). We used Hounsfield unit (HU) values at −29 to +150 HU to semi-automatically delineate muscle. Muscle area and density were quantified by SliceOmatic V4.3 software (Tomovision, Montreal, Canada). Mean paraspinal muscle density was measured to evaluate skeletal muscle fat content indirectly. According to previous study, the higher the muscle fat concentration, the lower the muscle density, skeletal muscle fat index (SMFI), was calculated as 100 *[Paraspinal muscle area (cm^2^)/paraspinal muscle density (HU)], to evaluate skeletal muscle fat content by CT-based paraspinal muscle density. Thus, and the higher the muscle fat content the higher the SMFI. All CT images were analyzed by one trained observer.

### Statistical Analysis

The participants were divided into 4 groups according to SMFI quartiles (Q1-Q4) as follows: Q1, ≤50.54 ± 6.89; Q2, 67.35 ± 3.84; Q3, 80.69 ± 4.03; Q4, ≥102.74 ± 14.69. Numerical data were expressed as mean ± standard deviation for normally distributed variables. Categorical variables are presented as percentage. One-way ANOVA analysis was applied to compare the means and Chi square test was applied to the comparisons of proportions. Correlation analysis was performed using Pearson’s correlation and Partial correlation. Logistic regression analysis was conducted to determine the correlations between SMFI quartiles and the risk of hyperuricemia, for quartiles 2-4, the odds ratio (OR) and 95% confidential interval (CI) of hyperuricemia were calculated and compared with the lowest quartile as the reference category. SPSS 26.0 (SPSS, Chicago, IL, USA) was used for statistical analyses. P values < 0.05 were considered statistically significant.

## Results

### General Characteristics of Participants With Different Quartiles of SMFI

Among the participants, 38.8% were women and 61.2% were men. **Table 1** showed the clinical characteristics of participants of quartile1(Q1) to quartile4 (Q4) of SMFI. Participants with higher quartiles of SMFI had a higher BMI, ALT, UA, Cr, TG (p<0.05) and a lower HDL-c (p<0.05) than participants in the lower quartiles of SMFI. The percentage of male participants was increased among quartiles of SMFI. The age of participants in Q2 was higher than that in Q1(p<0.05). There was no significant difference of BUN, TC, and LDL-c among different quartiles of SMFI.

**Table 1:** Characteristics of all subjects with different SMFI quartiles stratification.

	Q1 (n=125)	Q2 (n=125)	Q3 (n=125)	Q4 (n=125)	P value	*Post hoc*
**Age**	53.82 ± 13.77	59.42 ± 15.74	57.13 ± 13.19	57.23 ± 14.79	0.023	aa
**Male (%)**	21.6	60.8	74.4	88.0	<0.001	N/A
**BMI (Kg/m^2^)**	20.68 ± 2.85	23.05 ± 2.65	24.69 ± 2.58	27.30 ± 3.42	<0.001	aaa, bbb, ccc, ddd, eee, fff
**ALT**	15.82 ± 7.43	22.46 ± 18.00	24.26 ± 16.02	25.66 ± 13.01	<0.001	a, bbb, ccc
**AST**	18.64 ± 8.52	20.64 ± 8.56	20.67 ± 9.11	21.34 ± 7.60	0.07	c
**UA**	277.64 ± 71.38	325.7 ± 86.14	340.16 ± 85.58	385.86 ± 93.14	<0.001	aaa, bbb, ccc, eee, fff
**Cr**	53.19 ± 11.32	64.76 ± 14.24	67.70 ± 16.42	68.78 ± 15.81	<0.001	aaa, bbb, ccc
**BUN**	4.97 ± 1.57	5.11 ± 1.38	5.23 ± 1.27	5.30 ± 1.16	0.251	
**TG**	1.12 ± 0.75	1.54 ± 1.12	1.81 ± 1.15	2.06 ± 1.22	<0.001	aa, bbb, ccc, eee
**TC**	4.82 ± 0.92	4.66 ± 0.96	4.62 ± 1.03	4.79 ± 1.21	0.365	
**HDL-c**	1.41 ± 0.40	1.16 ± 0.33	1.10 ± 0.32	1.03 ± 0.27	<0.001	aaa, bbb, ccc, ddd, ee
**LDL-c**	2.79 ± 0.74	2.73 ± 0.82	2.63 ± 0.84	2.78 ± 0.84	0.426	
**SMFI**	50.54 ± 6.89	67.35 ± 3.84	80.69 ± 4.03	102.74 ± 14.69	<0.001	aaa, bbb, ccc, ddd, eee, fff

a: Q1 vs. Q2, b: Q1 vs. Q3, c: Q1 vs. Q4, d: Q2 vs. Q3, e: Q2 vs Q4, f: Q3 vs. Q4

1x, 2x and 3x letter = p <0.05; p <0.01 and p <0.001. All data are mean ± SD. N/A, not applicable.

### Correlations Between SMFI and Anthropometric and Metabolic Variables

Pearson’s correlation analysis showed SMFI was positively associated with BMI, TG, UA, Cr and ALT, and negatively associated with HDL-c. After adjusted for BMI, age and gender, only UA showed a positive association with SMFI (**Table 2**).

**Table 2:** Correlation of anthropometric and metabolic variables with SMFI.

	Pearson’s correlation		Partial correlation (BMI, age, gender adjusted)	
	r	P	r	P
**Age**	0.069	0.121	N/A	N/A
**BMI**	0.679	<0.001	N/A	N/A
**TC**	-0.017	0.698	0.026	0.571
**TG**	0.277	<0.001	0.013	0.779
**HDL-c**	-0.367	<0.001	0.009	0.845
**LDL-c**	-0.008	0.865	0.034	0.445
**UA**	0.426	<0.001	0.114	0.011
**Cr**	0.345	<0.001	0.069	0.124
**BUN**	0.066	0.139	0.011	0.812
**ALT**	0.235	<0.001	-0.053	0.239
**AST**	0.105	0.019	-0.066	0.142

N/A, not applicable.

### Logistic Regression Analyses for HUA and SMFI

**Table 3** showed the relationship between the quartiles of SMFI and HUA. The odds ratio for HUA was increased from Q2 to Q4, the risk of HUA in Q4 was 7.94-fold higher than the first quartile (95% CI 3.55-17.77, p<0.001). After adjusted for BMI, the association between SMFI and risk of HUA was attenuated but still significant, the OR for HUA was 2.79 (95% CI 1.18-6.59, p<0.05) in Q2, 2.41(95% CI 1.00-5.81, p<0.05) in Q3, and 2.63 (95% CI 1.03-6.72, p<0.05) in Q4.

**Table 3:** Logistic regression analysis to determine the association between SMFI quartiles and HUA.

		OR	95% CI	P
**Model 1**	Q1	1.00		
	Q2	4.03	1.75-9.27	0.001
	Q3	4.62	2.02-10.54	<0.001
	Q4	7.94	3.55-17.77	<0.001
**Model 2**	Q1	1.00		
	Q2	2.79	1.18-6.59	0.019
	Q3	2.41	1.00-5.81	0.049
	Q4	2.63	1.03-6.72	0.044

Model 1: unadjusted.

Model 2: adjusted for BMI.

### Prevalence of HUA According to Quartile of SMFI

**Figure 1:**
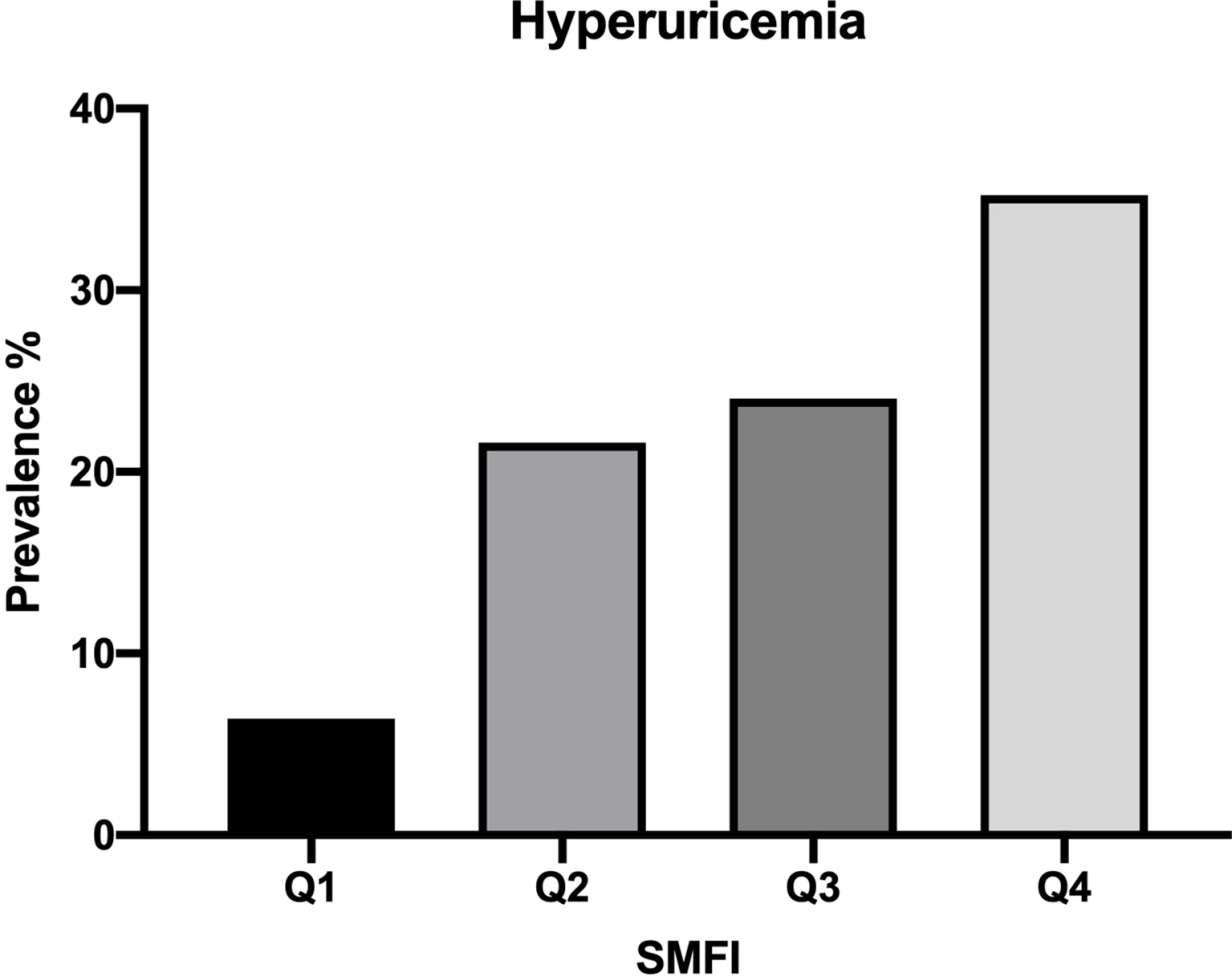
Prevalence of HUA according to quartile of SMFI.

**Figure 1** showed the prevalence of HUA was increased in sequence from the bottom to the top quartiles of SMFI (Q1:6.4%, Q2:21.6%, Q3:24.0%, Q4:35.2%, p<0.001).

## Discussion

It is the first study to evaluate the association of UA with muscle fat content. In the current study, we found that there was a significant association between SMFI and serum UA after adjusted for BMI, age and gender, and the subjects in the highest quartile of SMFI had 2.63-fold risk of hyperuricemia compared to the lowest quartile after adjusted for BMI.

It has been noticed that increased ectopic fat accumulations in skeletal muscle could affect body health, which is termed as myosteatosis. It’s well known that sarcopenia, which is characterized by decline of muscle mass and function, was observed as people aged. A longitudinal study suggested that increase in intermuscular adipose was a more consistent marker of aging than sarcopenia (23). Myosteatosis is also closely related to insulin resistance and has been reported as an independent risk factor of diabetes (19). It is indicated that the intramyocellular lipids content, which is analyzed by muscle biopsies, was significantly correlate with insulin resistance in T2DM patients, obesity as well as lean subjects (17). These findings confirmed the correlation between muscle fat content and metabolism, but uric acid was not analyzed in these studies. What’s more, studies also found the relationship between myosteatosis with frailty and decreased physical function. Studies conducted in the general population, hospitalized older patients and those with chronic illness found that myosteatosis was even associated with greater risk of mortality (24, 25).

Muscle fat content can be evaluated by invasive approach, such as muscle biopsy, and noninvasive approaches, such as computed tomography (CT), peripheral quantitative computed tomography (pQCT), magnetic resonance imaging (MRI) and quantitative ultrasound (QUS) (17). CT is one of the most widely used methods to evaluate fat content of muscle in relevant studies, which measures the muscle density by Hounsfield unit. Fat has a less dense in CT analysis, that is to say, the higher the content of fat, the lower the Hounsfield unit. It is more accessible and costs lower than other noninvasive approaches, and it has excellent reproducibility and reliability of muscle and adipose tissue attenuation. There were studies confirming the correlation of radiation attenuation of muscle with muscle fat content measured by biopsy. However, there is no standard method and value to define myosteatosis. Maxime et al. evaluate fat content of muscle by CT-based psoas density, and SMFI was calculated as 100*[psoas area (cm^2^)/psoas density (HU)]. They found SMFIPsoas was significantly associated with non-alcoholic fatty liver disease (NASH) even after adjusted for multiple confounders (26). In our study, we used paraspinal muscle density and calculated SMFI by 100 *[Paraspinal muscle area (cm^2^)/paraspinal muscle density (HU)], and it had a strong correlation with SMFIP_soas_ (**Figure S1**).

A positive association between skeletal muscle mass and/or muscle strength with UA level was found in many studies. A study enrolled 4236 adults aged 50 years above in China showed higher uric acid levels were significantly correlated with higher muscle mass and grip strength (27). A study on kidney transplant patients found that serum UA level was positively associated with muscle mass and handgrip strength (28). Xi et al. also found a positive association between serum UA level and appendicular skeletal muscle mass in patients on peritoneal dialysis (29). Dong’s study indicated the elevated serum UA was associated with a greater muscle mass in a middle-aged and elderly Chinese population (30). Roberta et al. also found skeletal muscle mass was directly related to UA level (31). Another study found that higher uric acid serum levels are associated with better muscle function in the oldest old (mean age 92.8 years) (16). In a study of children and adolescents, it showed that children in the highest quartile of muscle mass presented higher UA levels (32). Another study concluded that skeletal muscle index (SMI) and grip strength were positively associated with UA level in overweight and obese subjects (33). The limitation of the studies was that BMI or weight was not adjusted when the association was analyzed, which is both positively correlated with muscle mass and UA level in population. However, a study on US people aged 40 and above revealed that elevations in serum uric acid are significantly related to sarcopenia (13). A cross-sectional study showed a specific range of serum UA levels was correlated with grip strength positively (15). Esther et al. found there was an age difference in the association between UA and muscle strength: in adults aged 20 to 40 years, UA was negatively correlated with muscle strength, while after the age of 60, there was a positive correlation (34). The discrepancy may because of the variance of population, they are in different age and physical state, which may influence UA levels as well as muscle, and assessment of muscle is different in these studies, there were also differences in statistical analysis, variables included and adjusted in analysis also cause impact on the results. It also indicated that further analysis of the correlation between UA and muscle quality is necessary.

However, study evaluating the association of UA with muscle fat content is scarce. Michael et al. conducted an analysis on the correlation of skeletal muscle density and serum metabolomic and found decreased renal function (increased blood urea nitrogen, creatinine, uric acid) was linked with reduced muscle density (35), which is indirectly consistent with our results. The underlying mechanism is not fully elucidated yet. Studies have found the relationship between HUA and elevated serum lipid levels, which indicated the association between uric acid and lipid metabolism (7). There were some isozymes involved in purine metabolism have been isolated from muscle (36), and irisin, a novel hormone secreted by skeletal muscle, was associated with uric acid metabolism. What’s more, genome-wide association studies (GWAS) identify cell-type specific loci of skeletal muscle influencing serum uric acid (37, 38). The elevation of fat content in skeletaSl muscle may cause impact on gene and protein related to purine metabolism. These findings provide clues to further elucidate molecular mechanism of the link between skeletal muscle with serum UA homeostasis.

There were some limitations in the present study. First, it was a retrospective cross-sectional design and the sample size was relatively small, subjects were not divided into different gender, age and BMI groups for further analysis. Second, CT is not a direct measurement of fat content of skeletal muscle. Third, it did not control for potential biases from diet, physical activity, metabolic diseases and pharmacy history.

## Conclusions

The SMFI was significantly associated with the level of serum UA, and the higher SMFI may indicate a higher risk of HUA, independent of BMI. The results of this study provide valuable evidence for fat content of muscle in detecting HUA in the Chinese population. Therefore, a prospective study well-controlled and fundamental experiment research would be needed to verify the causal link between myosteatosis with UA.

## Data Availability Statement

The raw data supporting the conclusions of this article will be made available by the authors, without undue reservation.

## Ethics Statement

The studies involving human participants were reviewed and approved by Ethical Committee of Renji Hospital, School of Medicine, Shanghai Jiaotong University. Written informed consent for participation was not required for this study in accordance with the national legislation and the institutional requirements.

## Author Contributions

Conceptualization: NC and TH. Methodology: NC. Software: JC. Formal analysis: NC and TH. Investigation: HL, WL, DZ, TZ, and XL. Data curation: YW. Writing—original draft preparation: NC and TH. Writing—review and editing: YH. Supervision: XJ. Project Administration: YH. All authors contributed to the article and approved the submitted version.

## Funding

This study was supported by the National Natural Science Foundation of China (No.81870554) and the Foundation from Renji Hospital, School of medicine, Shanghai Jiaotong University (2019NYLYCP0102).

## Conflict of Interest

The authors declare that the research was conducted in the absence of any commercial or financial relationships that could be construed as a potential conflict of interest.

The reviewer NW declared a shared parent affiliation with the authors to the handling editor at the time of review.

## Publisher’s Note

All claims expressed in this article are solely those of the authors and do not necessarily represent those of their affiliated organizations, or those of the publisher, the editors and the reviewers. Any product that may be evaluated in this article, or claim that may be made by its manufacturer, is not guaranteed or endorsed by the publisher.
