# Supplementary material for: Muscle Fat Content Is Strongly Associated With Hyperuricemia: A Cross-Sectional Study in Chinese Adults
Source: Front Endocrinol (Lausanne). 2022 Jun 28;13:935445. doi: 10.3389/fendo.2022.935445 (PMC9275559; doi:10.3389/fendo.2022.935445)
Supplement: Supplementary file 1 [file DataSheet_1.docx]

Supplementary Material

# Supplementary Data

85 cases of the participants underwent an abdominal CT scan at level of third lumbar vertebra (L3) to assess of cross-sectional areas and muscle density of the psoas, SMFI_Psoas_ was calculated as 100 *[ psoas area (cm^2^)/ psoas density (HU)]. Pearson’s correlation analysis was employed for evaluating the correlation between L3- SMFI_Psoas_ and T12- SMFI_Paraspinal_. It showed that SMFI_Psoas_ was positively associated with SMFI_Paraspinal_. Scatter plot was also presented.

# Supplementary Figures and Tables

## Supplementary Figures


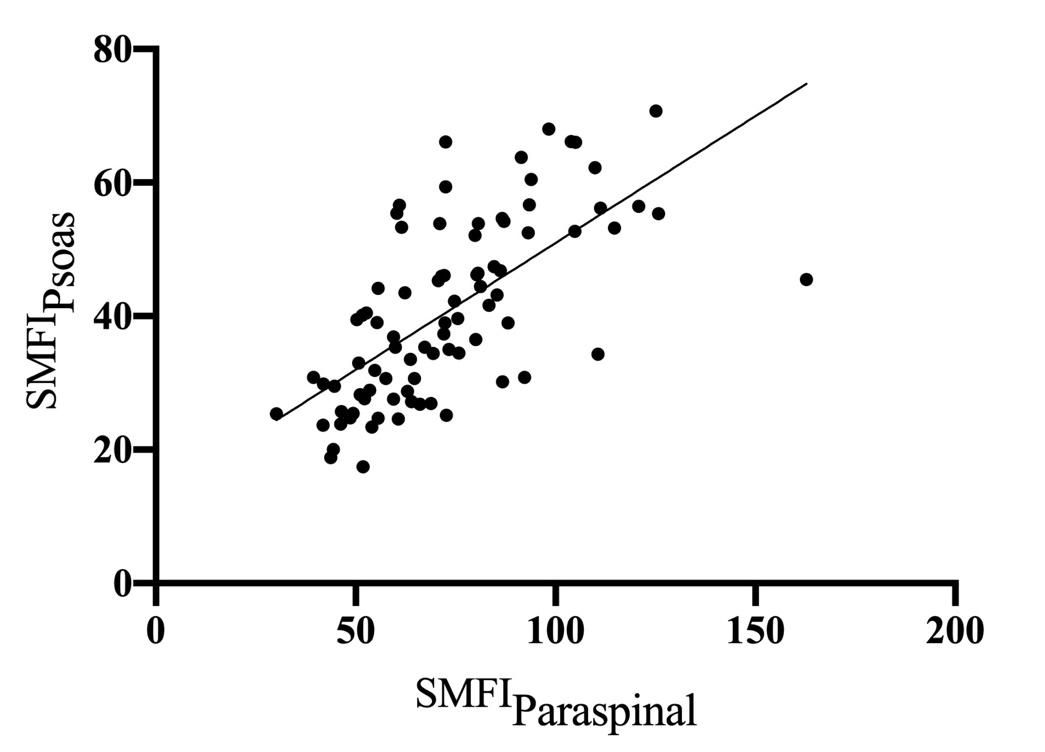


**Supplementary Figure 1.** Scatter plot of L3- SMFI_Psoas_ and T12- SMFI_Paraspinal_ in a subset of 85 cases.

## Supplementary Tables

| Pearson’s correlation | |
| --- | --- |
| **r** | ***p*** |
| **0.666** | **<0.001** |

**Supplementary Table 1.** Pearson’s correlation analysis between L3- SMFI_Psoas_ and T12-SMFI_Paraspinal_.
